# Cortical oxygen consumption in mental arithmetic as a function of task difficulty: a near-infrared spectroscopy approach

**DOI:** 10.3389/fnhum.2013.00217

**Published:** 2013-05-22

**Authors:** Martin Verner, Martin J. Herrmann, Stefan J. Troche, Claudia M. Roebers, Thomas H. Rammsayer

**Affiliations:** ^1^Department of Psychology, University of BernBern, Switzerland; ^2^Center for Cognition, Learning, and Memory, University of BernBern, Switzerland; ^3^Department of Psychiatry, Psychosomatics and Psychotherapy, University of WürzburgWürzburg, Germany

**Keywords:** near-infrared spectroscopy, mental arithmetic, task difficulty, oxygen consumption, cortical activation

## Abstract

The present study investigated changes in cortical oxygenation during mental arithmetic using near-infrared spectroscopy (NIRS). Twenty-nine male volunteers were examined using a 52-channel continuous wave system for analyzing activity in prefrontal areas. With the help of a probabilistic mapping method, three regions of interest (ROIs) on each hemisphere were defined: The inferior frontal gyri (IFG), the middle frontal gyri (MFG), and the superior frontal gyri (SFG). Oxygenation as an indicator of functional brain activation was compared over the three ROI and two levels of arithmetic task difficulty (simple and complex additions). In contrast to most previous studies using fMRI or NIRS, in the present study arithmetic tasks were presented verbally in analogue to many daily life situations. With respect to task difficulty, more complex addition tasks led to higher oxygenation in all defined ROI except in the left IFG compared to simple addition tasks. When compared to the channel positions covering different gyri of the temporal lobe, the observed sensitivity to task complexity was found to be restricted to the specified ROIs. As to the comparison of ROIs, the highest oxygenation was found in the IFG, while MFG and SFG showed significantly less activation compared to IFG. The present cognitive-neuroscience approach demonstrated that NIRS is a suitable and highly feasible research tool for investigating and quantifying neural effects of increasing arithmetic task difficulty.

## Introduction

Basic mental arithmetic bears a helping hand in many occasions of daily life. It is essential for time management, is a central aspect of mathematical achievement in school, but also supports us in many everyday life decisions as for instance in grocery stores. Many everyday situations involve encoding and manipulating of numerical information. Even in young children, mental arithmetic can be studied, for example, in the form of precursors of mathematical skills (e.g., counting, number sense; Gilmore et al., 2010), and the mastery of more and more complex mental arithmetic tasks in ontogeny provides a window for studying the constituents of these mental operations (Wynn, 1992; Geary, 1995; Lee et al., 2012; Van Der Ven et al., 2012). Thus, from a scientific point of view, mental arithmetic provides an ideal function for investigating fundamental cognitive processes such as retrieving information, execution of control processes, updating of information, and the like. In the present study, a cognitive-neuroscience approach is presented aiming at identifying and quantifying (in terms of blood oxygen consumption) neural correlates involved in the mastery of simple and more complex mental addition tasks.

Addition is one of the core operations in mental arithmetic. According to Van Harskamp and Cipolotti (2001) the simple addition of two numbers entails three specific processes: (1) retrieval of mathematical facts from long-term memory (e.g., 7 + 8 = 15), (2) the execution of the arithmetic operation as represented by an arithmetic symbol (e.g., “+”), and (3) the implementation of supporting arithmetic procedures like carrying. Carrying refers to decomposing the 8 into 3 and 5, bridging to ten, maintaining the first digit in working memory (i.e., updating), and then adding the remaining addend for completing the calculation. Studies on the development of arithmetic skills documented that there is a shift from premature arithmetic strategies observed in young children (e.g., “counting-on” or “using-the-fingers” strategies) to more sophisticated strategies as retrieval-based techniques or the decomposition strategy (Flynn and Siegler, 2007; Imbo and Vandierendonck, 2007). The use of the latter mirrors age-related improvements in the efficiency of mathematical problem solving (Siegler, 2006; Lemaire and Callies, 2009). When studying adults, therefore, the predominant strategy for simple arithmetic (SA) tasks (e.g., 12 + 6) will consist of fast retrieval-based techniques, while more complex tasks with large addends (e.g., 27 + 38) will be solved through a coordinated mix of retrieval-based techniques, decomposition, and updating processes (Seitz and Schumann-Hengsteler, 2002). The complexity of arithmetic tasks is mainly determined by the number of the necessary additional cognitive processes. For example, several studies indicated the number of carry operations in an addition task to be highly correlated with the time needed to solve this task (Ashcraft and Faust, 1994; Ashcraft and Kirk, 2001; Imbo et al., 2007). Other studies showed inefficient carrying to be a central cause of errors in mental arithmetic (Fürst and Hitch, 2000).

The main requirements for efficient carry operations are the ability to store interim results temporarily, the use of problem-solving skills, and the use of rule-based procedures. The resources necessary for these operations are absorbed primarily by working memory (Geary and Widaman, 1987). Complex arithmetic tasks require more working memory resources than SA tasks (Kong et al., 2005; Fehr et al., 2007) and are more strongly negatively affected by a secondary task, especially when the secondary tasks absorbs additional executive resources (Seitz and Schumann-Hengsteler, 2002). Traditional behavioral approaches to calculation skills, however, leave open the question concerning the relative importance of the different cognitive mechanisms involved in mental arithmetic. Neuroimaging studies may contribute to a better understanding of cognitive processes and brain mechanisms underlying mental arithmetic as will be outlined in the following paragraphs.

In an often-cited study on mental arithmetic (Ischebeck et al., 2006), reduced working memory load was accompanied by decreased brain activation in inferior frontal areas as measured by functional magnetic resonance imaging (fMRI). Other neuroimaging studies indicated highly complex cerebral networks to be involved in arithmetic task performance including the prefrontal cortex (PFC), cingulate cortex, fusiform gyrus, insula, cerebellum and the parietal cortex (for a review see Arsalidou and Taylor, 2011). More specifically, there is some evidence for the notion that the cingulate gyrus may coordinate and integrate activity of multiple attentional systems (Peterson et al., 1999). The fusiform gyrus seems to be crucial for encoding object properties (Allison et al., 1994) and visual number form (Dehaene and Cohen, 1997). The insula is associated with the execution of responses (Huettel et al., 2001), error processing (Hester et al., 2004), and, thus, might act as a network hub during information processing (Sridharan et al., 2008). The parietal cortex plays a crucial role in verbal number processing, quantity representation, and attentional processes (Dehaene et al., 2003). The cerebellum (Stoodley and Schmahmann, 2009) and PFC (Owen et al., 2005), are involved in working memory and executive functioning.

In a recent meta-analysis including 53 fMRI data sets on brain activation during mental arithmetic, Arsalidou and Taylor (2011) identified three distinct regions in the prefrontal cortex that contribute to performance on mental arithmetic. The inferior frontal gyri (IFG) were reported to be active during basic numerical tasks with only little storage requirements, while the middle frontal gyri (MFG) seemed to be involved in calculations entailing procedural steps like carrying. When the tasks contained multi-step problems or when computational strategies were required, activity in the superior frontal gyri (SFG) was observed.

The primary goal of the present study was to examine whether differences in arithmetic task complexity are associated with different levels of oxygen consumption in IFG, MFG, and SFG. While most previous studies used fMRI, our study contributes to the growing number of studies using near-infrared spectroscopy (NIRS). NIRS allows to measure changes in oxygenated (O_2_Hb) and deoxygenated hemoglobin (HHb) in the cortical surface of the human brain while subjects perform cognitive tasks. It is widely accepted that increases in O_2_Hb and decreases in HHb indicate cortical activation (Strangman et al., 2002a). Numerous studies documented a high correspondence between NIRS and other functional brain imaging techniques such as fMRI (e.g., Strangman et al., 2002b; Huppert et al., 2006; Eggebrecht et al., 2012) and provided empirical evidence for the reliability and validity of NIRS data (e.g., Plichta et al., 2006a,b; Sato et al., 2006; Schecklmann et al., 2008).

We performed a comprehensive literature search that resulted in four NIRS studies accentuating the role of the PFC during mental calculations (Tanida et al., 2004; Yang et al., 2009; Pfurtscheller et al., 2010; Power et al., 2010). While three of these studies (Tanida et al., 2004; Pfurtscheller et al., 2010; Power et al., 2010) did not systematically vary arithmetic task complexity, Yang et al. (2009) used only a small number of sampling. Most commonly, NIRS studies on mental arithmetic use visually presented tasks and, thus, do not necessarily generalize to many everyday-life situations where solving of verbally presented mental arithmetic tasks is required. Therefore, in analogue to many daily life situations, our participants listened to the arithmetic task, kept the information in mind, and gave the solution verbally.

## Materials and methods

### Participants

Twenty-nine German speaking men ranging in age from 20 to 28 years (mean age ± standard deviation: 23.2 ± 2.5 years) participated in the present study. In order to avoid unwanted variance due to potential gender differences in the functional and structural neuroanatomy of mathematical cognition (Keller and Menon, 2009), we decided to only include men in the present study. According to self-reports, all participants were right-handed and had no actual or past neurological or psychiatric disorder. Written informed consent was obtained from each participant after detailed explanation about the study protocol and the NIRS recording was given. Participants were recruited by online or placard announcements and were rewarded with €20.00 after completion of the experiment. The study was approved by the Ethics Committee of the Faculty of Human Science of the University of Bern.

### Procedure

#### Mental arithmetic task

***Apparatus and stimuli***. Mental arithmetic tasks were arithmetic addition tasks in the number domain from 1 to 99 presented acoustically via loudspeakers at an intensity of 60 dB. Presentation of the tasks was controlled by E-Prime Version 2.0 experimental software (Psychology Software Tools Inc., Pittsburgh, PA, USA). Presentation of a single trial lasted about 1.6–2.6 s.

***Task procedure***. The tasks were presented by a pre-recorded, standardized female voice through loudspeakers. The main reason for using a female voice to present the tasks was that in numerous studies women talkers were generally found to be more intelligible than men talkers (e.g., Bradlow et al., 1996; Markham and Hazan, 2004). Testing took place in a soundproof chamber. All participants were tested individually. During the testing session, the participant sat alone in the soundproof chamber, the experimenter was outside the chamber. Participant's responses were registered by the experimenter by means of an intercom system.

The experimental task consisted of three conditions. In the SA condition each trial contained one two-digit and one single-digit addend (e.g., 34 + 8), while in the complex arithmetic (CA) condition two 2-digit addends were presented (e.g., 34 + 57) with the first addend larger than the second in half the trials and the second larger than the first in the other half. To ensure that the tasks involve computational effort, in both conditions the sum of the last digits of both addends exceeded 10 in all tasks. However, complex carrying—in terms of decomposing *and* reassembling single addends—was required in the CA condition only.

In an active control condition, similar stimuli were presented as in the SA and CA conditions. The first addend, however, was exchanged for the letter “Y” (e.g., Y + 68) and the participant's task consisted of simply repeating the second addend to prevent any mental calculation while other processes, such as perceptual processing of the stimuli and verbal responses, were similar to the experimental conditions.

The task was divided into 12 blocks with four blocks for each condition. Each block lasted 40 s followed by a 40-s rest period to allow for the hemodynamic response function to return to baseline. The order of blocks was counterbalanced across participants. Within each block, trials were presented in a fixed order. Participants' responses were given verbally. Immediately after the response, the experimenter pressed one of two designated keys referring to a correct or incorrect response logging accuracy and latency of responses. The next trial started immediately after response logging. Participants were not given the option to correct an answer and they did not receive feedback. Two sample tasks for each condition were given prior to the experiment proper to familiarize participants with the tasks.

For each of the two experimental conditions, mean response time across all four blocks was computed for correct responses. In addition, the average number of correctly solved tasks per block and the mean hit rate across all blocks were calculated. Thus, hit rate was operationalized as the ratio of the number of correctly solved tasks to the total number of tasks presented within the SA and CA condition, respectively.

#### Near-infrared spectroscopy

**Figure 1:**
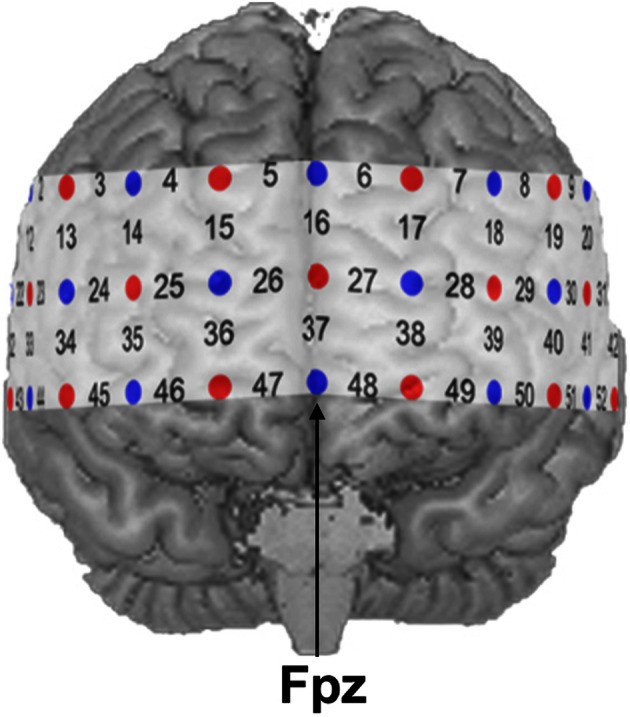
**NIRS probe set with 52 channels.** Emitters and detectors are represented by red and blue circles, respectively. The detector between channel positions 47 and 48 was placed over Fpz according to the 10–20 system.

NIRS measurements were conducted with an ETG-4000 Optical Topography System (Hitachi Medical Co., Japan) using a probe set consisting of 52 channels. A channel is defined as the region between one light emitter and one neighboring photo-detector. The 52 channels were divided into 17 emitters and 16 detectors building three rows with 11 optodes each (see Figure 1). Inter-optode distance was fixed at 30 mm resulting in measuring approximately 15–25 mm beneath the scalp. Two different wavelengths (695 ± 20 nm and 830 ± 20 nm) at a temporal resolution of 10 Hz were used. Changes of absorbed near-infrared light were transformed into relative concentration changes of O_2_Hb and HHb by means of a modified Beer-Lambert law. The unit of hemoglobin concentration was mmol × mm. Brain activity was indicated by an increase of O_2_Hb as well as a decrease of HHb (Strangman et al., 2002a; Obrig and Villringer, 2003). The probe set was placed over frontal regions with its central optode in the lowest row fixed over Fpz, while both ends of the probe set were placed symmetrically toward T3 and T4 according to the international 10–20 system for electroencephalography (Jasper, 1958).

To remove slow baseline drifts and high-frequency instrument noise in O_2_Hb and HHb, raw NIRS data were pre-processed. Using the software package ETG4000 V1.84eK (Hitachi Medical Co., Japan) a moving average filter with a time window of 5 s and a band pass filter with cut-off frequencies of 0.01 and 0.5 Hz were applied. To reduce additional spike-like noise in the continuous data (e.g., head motion artifacts) the signal was improved by applying a method based on the assumption that O_2_Hb and HHb are negatively correlated (Cui et al., 2010). This way, the data were also cleaned from potential artifacts due to the verbal responses given by the participants. Resulting data consisted of solely one parameter, i.e., a linear combination of O_2_Hb and HHb representing hemodynamic response. Larger values thereby indicate higher cortical activation.

In a first step, the mentioned linear combination of O_2_Hb and HHb was averaged for each of the 40-s blocks. This step included the subtraction of baseline-activation from task-activation values in each block. In a second step, the mean hemodynamic response for each condition was calculated by aggregating the single values from the first step across the four blocks. Finally, the resulting means in the simple and complex condition were corrected for the activation in the passive control condition by the subtraction method. The resulting values were considered indices of «pure» calculation-related cortical activation and were tested against 0 (*t*-tests as proof of activation). To avoid the risk of Type I error due to simultaneous testing, a false discovery rate approach (FDR; Singh and Dan, 2006) was applied. The FDR resulted in *p* and *t* values for SA, CA, and the comparison between SA and CA, respectively. In a second step, regions of interest (ROIs) were defined and corresponding channel positions were aggregated (see Results for more details).

To estimate accordance between channel positions and cortical topography and to make the data comparable with results provided by fMRI studies, a virtual registration procedure was used (Tsuzuki et al., 2007). This method utilizes structural information from an anatomical database (Okamoto et al., 2004; Jurcak et al., 2005) to provide estimates of the channel positions in a 3D reference frame (Montreal Neurological Institute coordinate system, MNI; Collins et al., 1994). This procedure also allows the estimation of spatial uncertainty due to intersubject variability of the channel positions. Thus, for each channel position the corresponding MNI-space coordinates with an estimated error was calculated (see Table 1).

**Table 1:** **Regions of interest (ROI), channel position numbers, MNI coordinates, and estimated inter-subject variability (SD)**.

**ROI**	**Channel**	**MNI-space correspondence**			
		***x***	***y***	***z***	***SD***
Left IFG	19	−55	18	31	5.8
	29	−52	36	19	7.1
	40	−56	28	7	5.4
	50	−51	44	−6	5.9
Right IFG	13	58	16	31	6.8
	24	54	36	20	4.0
	34	59	27	8	4.5
	45	53	43	−6	5.9
Left MFG	7	−30	41	44	4.8
	8	−46	24	44	4.6
	18	−42	42	32	5.7
	28	−35	58	20	5.5
	39	−44	53	6	3.2
	49	−35	64	−5	5.9
Right MFG	3	48	22	45	5.7
	4	33	40	44	5.7
	14	45	40	32	6.2
	25	38	57	20	4.8
	35	47	52	7	4.0
	46	38	63	−4	6.0
Left SFG	6	−10	52	45	5.1
	17	−22	57	33	5.7
	27	−13	68	20	5.4
	38	−24	68	8	4.8
	48	−13	71	−3	5.8
Right SFG	5	13	51	45	5.8
	15	25	57	32	6.2
	26	15	68	21	3.2
	36	27	68	8	4.4
	47	15	71	−3	5.4

## Results

### Behavioral data

Due to higher task difficulty, participants needed significantly more time to correctly solve a single arithmetic task in the CA than in the SA condition [*t*_(28)_ = −12.1; *p* < 0.001; *d* = −3.18]. Mean response times (± SEM) were 4270 ± 156 ms for the SA and 7244 ± 353 ms for the CA condition. Faster response times in the SA compared to the CA condition implied that participants were presented with a larger number of trials per block in the SA (9.91 ± 0.31) than in the CA (6.23 ± 0.31) condition [*t*_(28)_ = 20.5; *p* < 0.001; *d* = 5.38]. Average number of correctly solved trials per block was 9.28 ± 0.35 and 4.89 ± 0.32 for the SA and CA condition, respectively. As to accuracy of performance, the hit rate of 0.93 ± 0.01 observed under the SA condition was reliably higher than the hit rate of 0.77 ± 0.03 observed under the CA condition [*t*_(28)_ = 6.8; *p* < 0.001; *d* = 1.79]. Altogether, these results clearly indicate that our experimental manipulation of task complexity was successful.

### NIRS data

**Figure 2:**
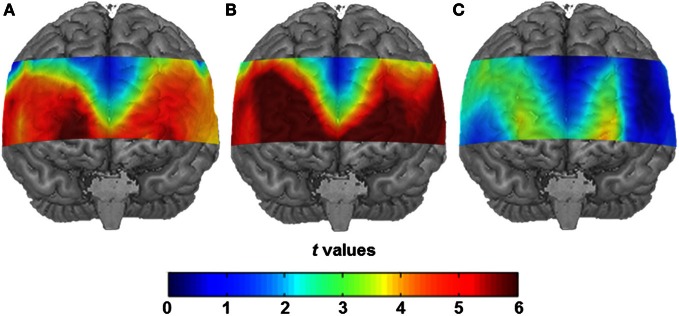
**(A)** Statistical activation map (*t*-values; *t*-test against 0) for hemodynamic response in the SA condition after correcting for the control condition. **(B)** Statistical activation map (*t*-values; *t*-test against 0) for hemodynamic response in the CA condition after controlling for the control condition. **(C)** Direct contrast of the two conditions (*t*-values, *t*-test between hemodynamic response in the SA condition and the CA condition).

Both conditions induced a significant task-related hemodynamic response. After correcting for simultaneous testing with the means of the FDR method (Singh and Dan, 2006), 43 channel positions yielded statistical significance in the SA condition (see Figure 2A) indicating cortical activation. Channel Positions 1, 2, 3, 4, 5, 6, 10, 16, and 17 failed to reach the 5% level of statistical significance. In the CA condition, the number of significantly active channel positions increased to 49 with only Channel Positions 5, 6, and 16 showing no significant activation (see Figure 2B). A direct comparison of the SA and the CA conditions indicated major differences in cortical activation to be located in the anterior prefrontal area. Reliably higher cortical activation was found in the CA compared to the SA condition (Channel Positions 12, 13, 14, 22, 24, 25, 35, 36, 46, and 47, in the left hemisphere, and Channel Positions 18, 28, 38, 39, 48, and 49, in the right hemisphere, respectively; see Figure 2C).

**Figure 3:**
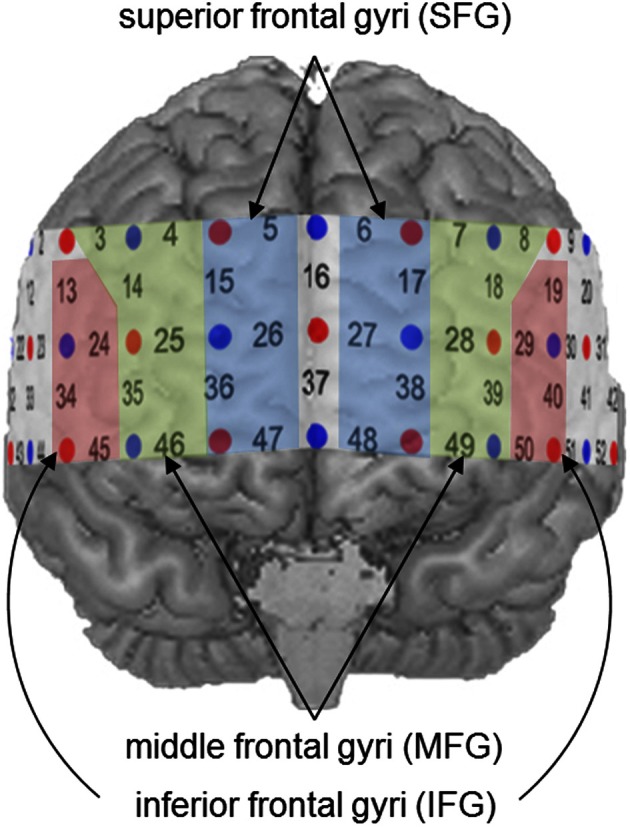
**Defined regions of interest (ROIs)**.

For further statistical analyses, specific ROIs were defined on the basis of previous findings. In a recent meta-analysis (Arsalidou and Taylor, 2011), the contribution of the prefrontal cortex during number and calculation tasks could be assigned to three distinct cortical regions. While the IFG were involved in the processing of simple numerical tasks, the MFG seemed to be active during cognitive procedural steps like carrying, and the SFG played a significant role in generating strategies during multi-step problems. Proceeding from these findings, all channel positions, likely to cover these prefrontal regions, were considered potential ROIs (see Figure 3). Activity in the IFG was measured by Channel Positions 19, 29, 40, and 50 (left hemisphere) as well as Channel Positions 13, 24, 34, and 45 (right hemisphere). The MFG were represented by Channel Positions 7, 8, 18, 28, 39, and 49 (left hemisphere) as well as Channels Positions 3, 4, 14, 25, 35, and 46 (right hemisphere). Finally, the SFG were covered by Channel Positions 6, 17, 27, 38, and 48 (left hemisphere) as well as 5, 15, 26, 36, and 47 (right hemisphere).

**Figure 4:**
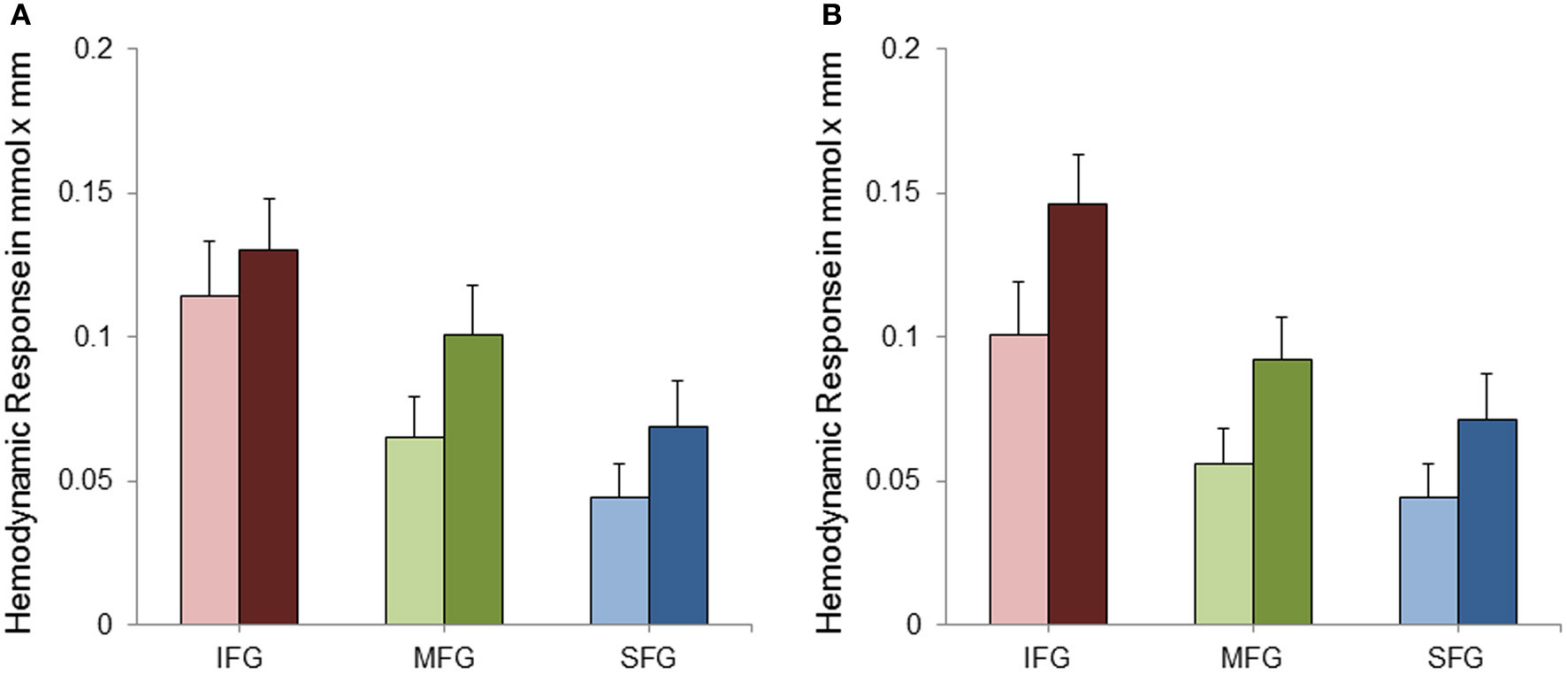
**Mean hemodynamic response and standard error of mean (SEM) for the left **(A)** and right **(B)** inferior frontal gyri (IFG), middle frontal gyri (MFG) and superior and medial frontal gyri (SFG) separately for the simple (SA; bright bars) and complex (CA; dark bars) mental arithmetic condition**.

Data was analyzed by means of a repeated measures analysis of variance (ANOVA) including three within-subject factors. These were (1) Hemisphere (two levels: right and left), (2) Task Complexity (SA and CA) and (3) ROI (IFG, MFG, and SFG). Greenhouse-Geisser corrected *p*-values are reported where appropriate (for all main effects of ROI and the interactions with ROI) to protect against violations of sphericity (Geisser and Greenhouse, 1958). Analysis of variance revealed a significant main effect of Task Complexity [*F*_(1, 28)_ = 9.22; *p* < 0.01; η^2^_p_ = 0.25] with the CA condition evoking a higher hemodynamic response than the SA condition (see Figure 4). As can also be seen from Figure 4, cortical activation decreased from inferior to middle and superior gyri [*F*_(1.15, 32.09)_ = 16.68; *p* < 0.001; η^2^_p_ = 0.37]. No statistically significant difference in cortical activation between the two hemispheres was observed [*F*_(1, 28)_ = 0.15; *p* = 0.70; η^2^_p_ = 0.01]. The interactions between ROI and Hemisphere [*F*_(1.38, 38.70)_ = 1.52; *p* = 0.23; η^2^_p_ = 0.05] and between ROI and Task Complexity [*F*_(1.05, 29.43)_ = 0.20; *p* = 0.67; η^2^_p_ = 0.01] did not reach statistical significance. The interaction between Task Complexity and Hemisphere yielded statistical significance [*F*_(1, 28)_ = 4.53; *p* < 0.05; η^2^_p_ = 0.14]. The effects mentioned so far, however, were modified by a significant three-way interaction [*F*_(1.15, 32.13)_ = 5.22; *p* < 0.05; η^2^_p_ = 0.16]. To further analyze this interaction a, *post-hoc* Scheffé test was applied. As to task complexity, the hemodynamic response increased significantly from the SA to the CA condition in all ROIs [right IFG (*p* < 0.001), left and right MFG (*p* < 0.001), left SFG (*p* < 0.05), right SFG (*p* < 0.01)] except for the left IFG (*p* = 0.47). With regard to the comparison of the three ROIs, IFG showed a more pronounced hemodynamic response in both conditions as well as both hemispheres [*p* < 0.001; for the SA condition, left hemisphere (*p* < 0.01)] compared to MFG. In the SA condition, the left MFG was more active than the left SFG (*p* < 0.001), all other contrasts between MFG and SFG did not yield statistical significance. For none of the three ROIs cortical activation differed between right and left hemisphere—neither in the SA condition [IFG (*p* = 0.84), MFG (*p* = 0.97), SFG (*p* = 1.0)] nor in the CA condition [IFG (*p* = 0.47), MFG (*p* = 0.97), SFG (*p* = 1.0)].

As noted above, most of the channel positions outside our ROI also yielded significant changes in hemodynamic response during task presentation. Most importantly, however, the majority of these channel positions did not differ in cortical activation between the SA and the CA condition. The only channel positions being sensitive for task complexity outside the defined ROI represented Channel Positions 12 and 22.

In order to provide a control region, the channel positions covering different gyri of the temporal lobe were pooled (right hemisphere: 32, 33, 43, and 44; left hemisphere: 41, 42, 51, and 52). After aggregation, these channel positions did not show any significant differences in hemodynamic response as a function of task complexity [right hemisphere: *t*_(28)_ = 1.65; *p* = 0.11; left hemisphere: *t*_(28)_ = 0.99; *p* = 0.33]. In other words, the sensitivity to arithmetic task complexity appeared to be restricted to the specified ROIs.

## Discussion

In the present study, changes in prefrontal cortical blood oxygenation during mental arithmetic were quantified by means of NIRS. To our knowledge, this was the first NIRS study on mathematical processing using verbally presented arithmetic tasks that additionally varied in task complexity. The implemented addition tasks required mental effort and the coordination of different mental operations (in a certain sequence), including different arithmetic strategies (carrying, retrieval) as solutions could not be directly retrieved from memory. While the SA tasks required only a simple carry operation with one single digit addend and could be solved without temporarily storing the intermediate total in working memory, CA tasks demanded more computational effort since addends consisted of two digit numbers. Consistent with numerous studies investigating mental arithmetic (Ashcraft and Faust, 1994; Fürst and Hitch, 2000; Ashcraft and Kirk, 2001; Imbo et al., 2007), our tasks requiring the more complex carry operation led to more calculation errors compared to the SA tasks, and participants needed more time to solve the CA compared to the SA tasks. The higher number of calculation errors and the longer response times in the CA than in the SA condition indicated that our manipulation of task complexity had been successful.

As to overall blood oxygen consumption, both mastery of the simple and the complex arithmetic problems was associated with reliable increases of brain activation as reflected by an increase of oxygen consumption from the control to the two experimental conditions. Furthermore, activation of mainly anterior prefrontal areas was higher in the CA compared to the SA condition indicating that NIRS sensitively displays changes in invested mental effort due to increased arithmetical task difficulty. Although in the SA condition more trials were presented than in the CA condition, oxygen consumption was higher in the CA compared to the SA condition.

Against the background of Arsalidou and Taylor's (2011) finding that IFG, MFG, and SFG serve different arithmetical functions, we proceeded from their assumptions and defined corresponding ROIs of the prefrontal cortex. In both task conditions and both hemispheres, the highest activation of all three ROIs was observed in the IFG emphasizing their important role in mental arithmetic. In their meta-analytic fMRI study, Arsalidou and Taylor (2011) proposed inferior prefrontal regions to play a crucial role in the processing of information requiring (rule-like) simple cognitive operations. Also in previous studies, IFG has been linked to task difficulty (Zhou et al., 2007), working memory, and attention (Ischebeck et al., 2009). Our data are partially consistent with Zhou et al.'s (2007) interpretation of IFG activation reflecting task difficulty as it increases from the SA to the CA condition (but see Kong et al., 2005) in the right IFG. However, *post-hoc* analyses indicated that task complexity did not influence activation of the left IFG suggesting task difficulty being somewhat lateralized to the right IFG.

As to the MFG, our finding of increasing bilateral activation in MFG with increasing complexity of the arithmetic tasks is in line with previous findings (cf., Arsalidou and Taylor, 2011). Activity in MFG was lower than in IFG and virtually equal to activity in SFG. Most interestingly, only in the CA condition, the left MFG was significantly more active than the left SFG indicating a large increase in cortical activation from the SA to the CA condition. MFG corresponds roughly to the dorsolateral prefrontal cortex and is typically associated with working memory functions (Owen et al., 2005). These dorsolateral areas are involved when coordination of subprocesses and cognitive control becomes more and more important (Rypma et al., 1999). As outlined above, our CA tasks required more coordination and cognitive control than the SA tasks. This may account for the bilaterally increasing MFG activity from the SA to the CA condition.

Compared to IFG and, at least in part, also to MFG, lower activation was measured in SFG. This lower activation might be attributed to the fact that channel positions covering more posterior parts of the SFG did not show any cortical activity at all (Channel Positions 5, 6, and 16). The activation of the SFG can largely be ascribed to further anterior parts of the SFG, namely to channel positions covering the anterior prefrontal cortex. The anterior prefrontal cortex, has been described as being active during goal-oriented coordination of different cognitive sub-operations (Ramnani and Owen, 2004). Because solving of arithmetical problems commonly requires more than one operation (Fürst and Hitch, 2000) and because more complex tasks yield more operations to be coordinated, the increase of activation in SFG from the SA to the CA condition is likely to reflect the higher task demands induced by the CA compared to the SA tasks.

It should be noted that the present activation patterns are not identical across all channel positions of the probe set. For the channels outside the defined ROIs, complexity-related increase of brain activation could not be observed except for Channels 12 and 22 (see also Figure 2C). Consequently, when channel positions covering different structures of the temporal lobe were combined to a control region of sorts, these temporal areas of the probe set were not sensitive to task complexity. The sensitivity to task complexity, therefore, seems to be restricted to the specific ROIs defined (i.e., IFG, MFG, SFG).

While Arsalidou and Taylor (2011) refer to a hemispheric asymmetry in parietal and prefrontal regions with addition being left lateralized, in the present study no indication of a lateralization effect could be observed, except for a higher sensitivity to task complexity for the right compared to the left IFG. When we compared single mean values of the hemodynamic response across hemispheres by means of indices of laterality as suggested by Binder et al. (1996), lateralization effects were found neither with SA nor with CA tasks. It is important to note, however, that our experimental design differed from the ones employed in previous studies in some important points. The verbal presentation of the arithmetic tasks in the present study could have led to increased cognitive load and, thus, hamper the comparison with previous studies which mainly used visually presented tasks. Furthermore, our participants had to additionally transform auditory information into a symbolic representation and to remember the initial addends during the entire calculation process. Because our design did not control for these processes, it might be possible that cortical activation due to “pure” calculation had been covered by such additional transformation and memory processes. Casasanto (2003) stated that left lateral prefrontal activation is linked to the verbalizability of non-verbal stimuli, whereas activation in right lateral prefrontal areas is related to the imageability of verbal stimuli. This imageability component might represent the very difference between the present study and previous work. At the same time, however, verbally presented mental arithmetic tasks bear the advantage of higher ecological validity.

Compared to other brain imaging techniques, NIRS has a few limitations such as a relatively low spatial resolution as compared to fMRI or positron emission tomography. NIRS measurements are also restricted to surface cortical areas only. Nevertheless, it depends on the research question to what extent the latter constitutes, in fact, a disadvantage. Potential disadvantages of NIRS, however, may be outweighed by practical features such as the provision of many more research possibilities (with higher ecological validity), the low sensitivity to movement artifacts, and low procedural and maintenance costs.

In sum, with NIRS as a research tool, the present study was able to corroborate the general significance of the prefrontal cortex for mastery of mental addition tasks. With the help of virtual registration methods, an approximate accordance between channel positions and cortical topography was achieved and activation patterns documented in the present approach were linked to findings of previous fMRI studies. More specifically, the increased necessity of cognitive control and the coordination of different sub-operations when addition tasks became more complex could be confirmed in terms of increased blood oxygen consumption in MFG and SFG that were found to be particularly sensitive to enhanced task difficulty. Thus, NIRS technology turned out to represent a highly feasible and non-invasive research method for investigating neural correlates of mental arithmetic. Based on the present findings, in future studies employing NIRS technology, preferential consideration should be given to the identification of different cognitive processes involved in the processing of SA and CA tasks. Furthermore, particular attention should be paid to the functional distinction between the frontal brain regions (IFG, MFG, and SFG) that were shown in Arsalidou and Taylor's (2011) meta-analyses of fMRI studies to be associated with different cognitive functions in mental arithmetic.

### Conflict of interest statement

The authors declare that the research was conducted in the absence of any commercial or financial relationships that could be construed as a potential conflict of interest.
